# Quantitative radiologic criteria for the diagnosis of lumbar spinal stenosis: a systematic literature review

**DOI:** 10.1186/1471-2474-12-175

**Published:** 2011-07-28

**Authors:** Johann Steurer, Simon Roner, Ralph Gnannt, Juerg Hodler

**Affiliations:** 1 Horten Centre for patient oriented research and knowledge transfer, University Zurich, Raemistrasse 100, CH 8091 Zurich, Switzerland; 2 Diagnostic and Interventional Radiology, University Hospital Zurich, Raemistrasse 100, CH 8091 Zurich, Switzerland

## Abstract

**Background:**

Beside symptoms and clinical signs radiological findings are crucial in the diagnosis of lumbar spinal stenosis (LSS). We investigate which quantitative radiological signs are described in the literature and which radilogical criteria are used to establish inclusion criteria in clincical studies evaluating different treatments in patients with lumbar spinal stenosis.

**Methods:**

A literature search was performed in Medline, Embase and the Cochrane library to identify papers reporting on radiological criteria to describe LSS and systematic reviews investigating the effects of different treatment modalities.

**Results:**

25 studies reporting on radiological signs of LSS and four systematic reviews related to the evaluation of different treatments were found. Ten different parameters were identified to quantify lumbar spinal stenosis. Most often reported measures for central stenosis were antero-posterior diameter (< 10 mm) and cross-sectional area (< 70 mm^2^) of spinal canal. For lateral stenosis height and depth of the lateral recess, and for foraminal stenosis the foraminal diameter were typically used. Only four of 63 primary studies included in the systematic reviews reported on quantitative measures for defining inclusion criteria of patients in prognostic studies.

**Conclusions:**

There is a need for consensus on well-defined, unambiguous radiological criteria to define lumbar spinal stenosis in order to improve diagnostic accuracy and to formulate reliable inclusion criteria for clinical studies.

## Background

Spinal lumbar stenosis is the most frequent indication for spine surgery in patients older than 65 years of age [1]. In clinical medicine lumbar spinal stenosis is defined as "buttock or lower extremity pain, which may occur with or without low back pain, associated with diminished space available for the neural and vascular elements in the lumbar spine"[2]. This definition includes two aspects: morphological abnormalities and clinical manifestations, neurogenic claudication, caused by the somatic anomaly.

From a radiological perspective, emphasizing the underlying structural anomaly, *stenosis of the spinal canal with or without clinical manifestations *is a more appropriate definition. The condition underlying the clinical manifestations, as the term implies, is a stenosis of the spinal canal and it is well known that not all patients with a narrowing of the spinal canal, verified by an imaging procedure, suffer from neurogenic claudication [3-6].

Proper research in patients with a particular illness requires a precise definition of the illness at issue - preferentially the underlying somatic anomaly - in order to formulate sensible and reliable inclusion criteria [7]. In a recent review Genevay [8] reported that researchers used quite different combinations of symptoms, clinical signs and radiological criteria to set up inclusion criteria for trials in patients with lumbar spinal stenosis. Imprecise definitions limit the interpretability and clinical relevance of trial results. In the case of lumbar spinal stenosis radiologic criteria are as relevant as clinical signs to characterize these patients. The North American Spine Society states in their guideline that imaging is the key noninvasive test for lumbar spinal stenosis, but they provide no radiological criteria for stenosis in these guidelines [2].

In the present study radiological criteria were collected that were published in the literature to describe and quantify lumbar spinal stenosis and we investigated which radiological signs have been used to establish inclusion criteria for prognostic studies in patients with this disorder.

## Methods

### Literature search

A three-step literature search was performed to identify potentially fitting studies. The search was performed by an experienced librarian (MG) with special training and skills in literature search. In a first step she searched in Medline and Embase (from 1974 to July 2010) for studies potentially reporting on radiological criteria for describing lumbar stenosis. Search was performed including the following MESH terms: "spinal stenosis", "lumbar vertebrae", "Magnetic resonance imaging", "Tomography X-Ray Computed" and "sensitivity and specificity". The search was restricted to English and German language. The search strategy for Medline is shown in additional file 1.

In a second phase the reference lists of papers identified in the first search was scanned for further publications about radiological signs of lumbar stenosis.

In a third phase systematic reviews were searched in Medline (from 1974 to July 2010) and the Cochrane Library related to prognostic issues in patients with lumbar spinal stenosis. Search was performed including the following terms: "spinal stenosis", "lumbar vertebrae", "treatment", and "systematic review". Original studies included in the systematic reviews were ordered in paper form. The rationale for evaluating original studies is the expectation that eligibility and inclusion criteria are described precisely, including precise radiological criteria, in studies fulfilling the quality criteria to be included in a systematic review.

### Eligibility criteria

Only studies reporting on preoperative imaging, using magnetic resonance imaging (MRI), computer tomography (CT), conventional myelography, or computer tomography-myelography (CT-myelography) were included if they described at least one radiological sign of spinal stenosis in quantitative terms. Conventional radiography is not an imaging modality of first choice in the diagnosis of lumbar spinal stenosis and studies on this method were not included in the review.

### Data extraction

Data extraction involved reviewing the method section to identify radiological criteria by two reviewers independently (SR and JS). Bibliographic information, details about the radiological method (MRI, CT, myelography, CT myelography) site of measurement (distances, areas, angles) and cut-off values for defining stenosis were extracted in a purpose defined form. We extracted data describing stenosis quantitatively.

### Statistical methods

Data are presented in a descriptive way. Additional statistical analysis was not performed.

## Results

**Figure 1:**
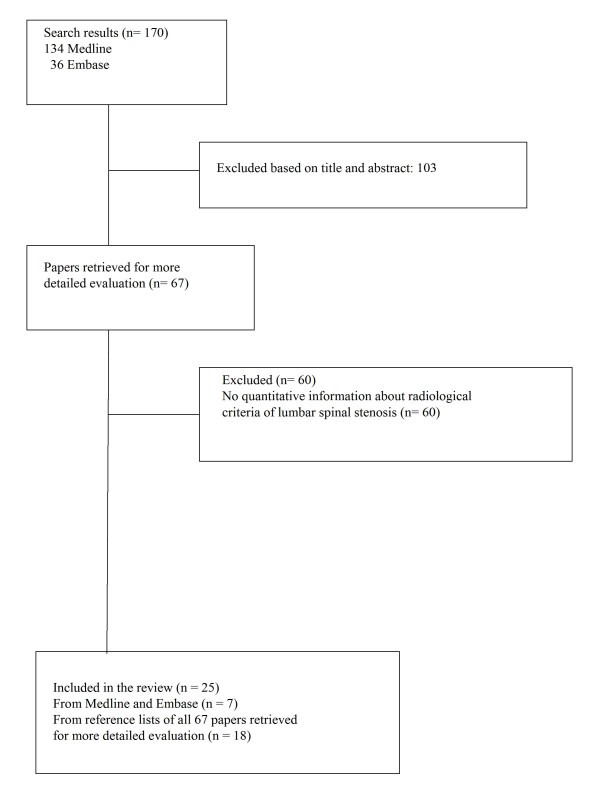
**Flowchart of search results in Medline, Embase and bibliographies**.

The search in Medline and Embase, described as step one, yielded, after excluding duplicates, 170 publications. After reading title and abstract 103 papers were excluded. For the remaining 67 publications full text versions were ordered. After reading the publications 7 papers fulfilled the inclusion criteria, and 60 papers were excluded because they did not report on any quantitative radiological sign of lumbar spinal stenosis. Based on the reference lists of the included papers 18 further papers were identified that were eligible for inclusion in the structured literature review (in total 25). Flowchart is shown in Figure 1.

In Medline and the Cochrane Library four systematic reviews including 63 original studies were identified [8-11]. Only 4 of the 63 original articles included patients with lumbar spinal stenosis and reported on quantitative radiological details in defining eligibility criteria. Three primary studies were included in more than one systematic review [12-14].

In the included studies, ten various parameters were applied with regard to lumbar stenosis. These measures might be distinguished into descriptors for central, lateral and foraminal stenosis, respectively.

### Descriptors for central stenosis

**Figure 2:**
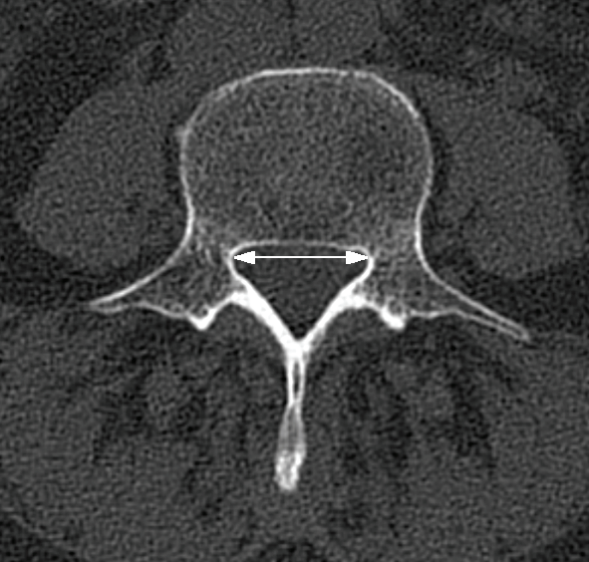
**Transaxial computed tomography image of the lumbar spine at the level of L4**. The white arrow indicates the transverse diameter of the osseous spinal canal.

**Figure 3:**
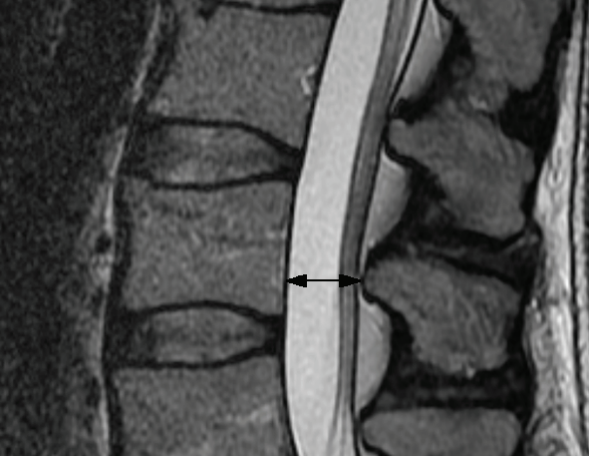
**T2 weighted sagittal fast spin echo MR image of the middle lumbar spine**. The black arrow indicates the antero-posterior diameter of the osseous spinal canal.

**Figure 4:**
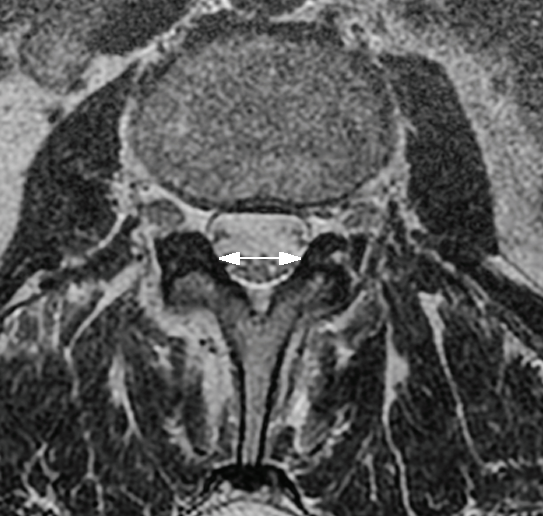
**T2 weighted transaxial fast spin echo MR image of the lumbar spine at the level of L3**. The white arrow indicates the ligamentous interfacet distance measured between the inner surfaces of flaval ligaments on a line connecting the joint space of facet joints.

**Figure 5:**
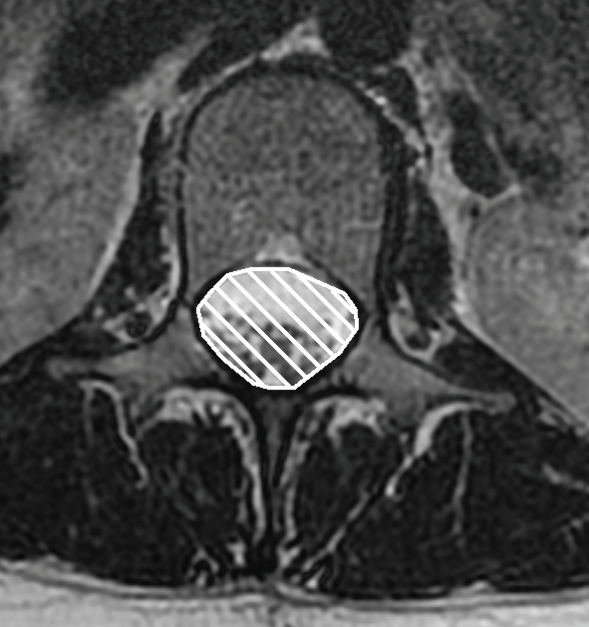
**T2 weighted transaxial fast spin echo MR image of the lumbar spine at the level of L1**. Cross sectional area of the spinal canal is indicated by the white hatched area.

For all methods, myelography, CT myelography, CT or MRI, different measurements are reported: Transverse (Figure 2) and antero-posterior (Figure 3) diameter of the osseous spinal canal, ligamentous interfacet distance (Figure 4) (distance between the inner surface of flaval ligaments on a line connecting the joint space of the facet joints at the level of the intervertebral disc), and cross sectional area of the spinal canal (Figure 5) [15]. Distances or areas were reported either in absolute numbers or in relative changes compared to specified reference measurements. Values for the antero-posterior diameter of the osseous spinal canal were reported and stenosis is defined by some authors by a distance of less than 10 mm, by others below 7 mm. In the majority most papers reporting a cross sectional area of the dural sac of less than 100 mm^2 ^indicated central stenosis. Detailed quantitative information about descriptors of central spinal stenosis is given in table 1.

**Table 1:** Sites of measurement, measurement points and radiologic definitions for central lumbar spinal stenosis

Imaging method	Author	Site of measurement	Level, where measured (measurement points)	Definition of stenosis (cut-off values)
MRI				

		*Antero-posterior diameter of spinal canal*		

	Fukusaki [22]		Not reported	< 15 mm

	Koc [23]		Not reported	< 12 mm

		*Mid-sagittal diameter of thecal sac*		

	Herzog [15]		Midbody of each vertebra	Compression of thecal sac area in % of normal mid-sagittal diameter: Grade 1: anterior < 15% posterior < 10% Grade 2: anterior 15 - 30% posterior 10 - 20% Grade 3: anterior > 30% posterior > 20%

		*Cross-sectional area of dural tube or sac*		

	Hamanishi [24]		Intervertebral levels: L2/3, L3/4, L4/5	< 100 mm^2^, at more than two of three intervertebral levels

	Mariconda [25]		Not reported	< 130 mm^2^

	Laurencin [26]		Motion segment: Intervertebral disc level coincident with flexible joint; Stable segment: Level coincident with the mid-pedicle unaffected by stenosis	Stenosis ratio: Cross-sectional area of dural sac of motion segment divided by stable segment cross-sectional dural sac area: Level: L3-L4 < 0.66 L4-L5 < 0.62 L5-S1 < 0.73

		*Ligamentous interfacet distance*		

	Herzog [15]		Distance between the innner surface of flaval ligaments on a line connecting the joint space of facet joints at the level of the intervertebral disc	< 10 mm (L2 - L3) < 10 mm (L3 - L4) < 12 mm (L4 - L5) < 13 mm (L5 - S1)

		*Transverse diameter of spinal canal*		

	Koc [23]		Not reported	< 15 mm

	Ullrich [27]		4 zones of measurement: upper, middle, lower zone of vertebral body and disk space	< 16 mm

				

CT				

		*Antero -posterior diameter of spinal canal*		

	Bolender [28]		5 mm intervals from L2 to L5	< 13 mm

	Haig [4]		Not reported	≤ 11.95 mm

	Lee [29]		Not reported	< 15 mm (suggesting narrowing) < 10 mm (usually diagnostic)

	Ullrich [27]		Four zones of measurement: Upper, middle, lower zone of vertebral body and disk space	< 11.5 mm

	Verbiest [30]		Not reported	< 12 mm (relative) < 10 mm (absolute)

		*Antero-posterior diameter of dural sac*		

	Kalichman [3]		Midvertebral body level	10 - 12 mm (relative) < 10 mm (absolute)

	Herzog [15]		Midbody of each vertebra	Compression of thecal sac area in % of normal mid-saggital diameter: Grade 1: anterior < 15% posterior < 10% Grade 2: anterior 15 - 30% posterior 10 - 20% Grade 3: anterior > 30% posterior > 20%

	Jönsson [31]		Disc level	≤ 10 mm

		Cross-sectional area of dural sac		

	Bolender [28]		5 mm intervals from L2 to L5	100 - 130 mm^2 ^(early stenosis) < 100 mm^2 ^(present stenosis)

	Laurencin [26]		Motion segment: Intervertebral disc level coincident with flexible joint stable segment: Level coincident with the mid-pedicle unaffected by stenosis	Stenosis ratio: Area of motion segment divided by stable segment area Level: L3-L4 < 0.66 L4-L5 < 0.62 L5-S1 < 0.73

	Schönström [32]		On each CT scan slice	< 100 mm^2^

	Schönström [33]		Not reported	75 - 100 mm^2 ^(moderate) < 75 mm^2 ^(severe)

	Ullrich [27]		4 zones of measurement: Upper, middle, lower zone of vertebral body and disk space	< 145 mm^2^

				

		*Ligamentous interfacet distance*		

	Herzog [15]		Intervertebral disc level	< 10 mm (L2-L3) < 10 mm (L3-L4) < 12 mm (L4-L5) < 13 mm (L5-S1)

	Wilmink [34]		Pedicular, infrapedicular and/or disc level	< 11 mm (L4-L5)

				

Myelography				

conventional		*Antero-posterior diameter of contrast column*		

	Airaksinen [35]		Narrowest point	> 12 mm 10 - 12 mm < 10 mm Subtotal block Total block

	Bolender [28]		Intervertebral level	< 13 mm

	Herno [36]		Not reported	< 12 mm

	Jönsson [31]		Disc level	≤ 10 mm



	Sortland [37]		Disc level	< 10.5 mm (lower limit) < 5.5 - 7 mm (considerable)

	Verbiest [38]		Superior and inferior boarders of the laminae	10 - 12 mm (relative) < 10 mm (absolute)

Myelo-CT	Mariconda [25]		Not reported	< 130 mm^2^

### Descriptors for lateral stenosis

**Figure 6:**
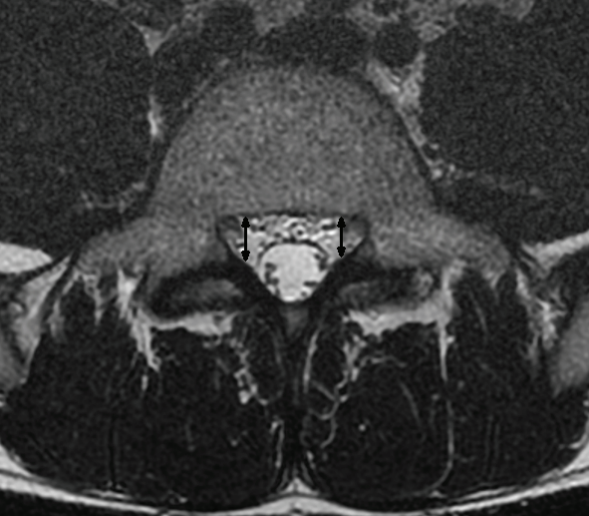
**T2 weighted transaxial fast spin echo MR image of the lumbar spine at the level of L5**. The depth of the lateral recess is measured between the superior articular facet and the top part of the pedicle marked with the black arrow.

**Figure 7:**
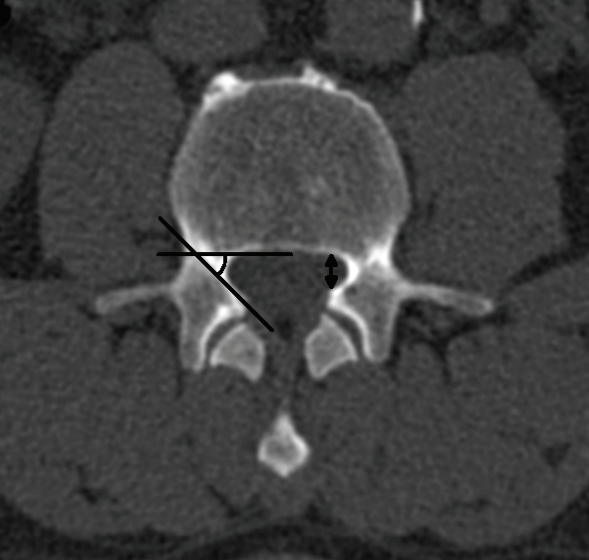
**Transaxial computed tomography image of the lumbar spine at the level of L3**. Left side: The lateral recess angle is defined as the angle between the lines parallel to the floor and the roof of the lateral recess. Right side: The height of the lateral recess is defined as the shortest distance from the most anterior point of the superior articular process to the posterior border of the vertebral body.

Height and depth of the lateral recess, and lateral recess angle are criteria to describe lateral stenosis. The depth of the lateral recess is measured between the superior articular facet and the top part of the pedicle (Figure 6). Recess height is specified as distance between the most anterior point of the superior articular facet and the posterior border of the vertebral body and the lateral recess angle as the angle between the lines parallel to the floor and the roof of the lateral recess (Figure 7) [16]. A lateral recess height ≤ 2 mm and/or lateral recess depth ≤ 3 mm or a lateral recess angle < 30° has been described as diagnostic for lateral recess stenosis. Detailed information is given in table 2.

**Table 2:** Sites of measurement, measurement points and radiologic definitions for lateral lumbar spinal stenosis

Imaging method	Author	Site of measurement	Level, where measured	Definition of stenosis (cut-off values)
CT		*Lateral recess height*		

	Ciric [39]		Not reported	5 mm (normal) ≤ 3 mm (highly indicative) ≤ 2 mm (diagnostic)

				

	Strojnik [16]		between the most medial point of the superior articular facet and the posterior point of the vertebral body	≤ 3.6 mm

				

		*Depth of lateral recess*		

	Dincer [40]		Between superior articular facet and the top part of the pedicle.	> 5 mm (normal) 4 - 5 mm (Group 3) 3 - 4 mm (Group 2) 2 - 3 mm (Group 1)

	Mikhael [41]		Between the posterior surface of the vertebral body and the anteromedial portion of the superior articular facet at the level of the superior border of the corresponding level	> 5 mm (normal) 3 - 5 mm (suggestive) ≤ 3 mm (definitive)

		*Lateral recess angle*		

	Strojnik [16]		Between the bottom and the roof of the triangular space (= lateral recess)	< 30°

### Descriptors for foraminal stenosis

The only quantitative criterion was the diameter of the foramen. A diameter of 2 to 3 mm is considered to indicate stenosis [17].

### Descriptors applied in primary prognostic studies, including surgery and drug treatment

Only in four of 20 primary studies [14,18-20] detailed radiological information for spinal stenosis was provided. Applied quantitative criteria for inclusion of patients in clinical studies were: diameter of spinal canal [14,19-21] and cross-sectional area of the dural tube [18,21]. Criteria for description applied in studies investigating patients with spinal stenosis are given in additional file 2.

## Discussion

The result of this literature review documents a remarkable list of various quantitative radiologic criteria applied to describe lumbar spinal stenosis. Measurement of antero-posterior diameter and the cross sectional area of spinal canal with varying cut-off levels are the most often applied criteria for central stenosis; height and length of the recess for lateral stenosis and foraminal diameter for foraminal stenosis. Only in a minority of primary prognostic studies, included in systematic reviews evaluating different treatment modalities, distinct and reliable criteria were used to set up eligibility criteria for patients included in these studies.

To our knowledge no structured and systematic review collecting radiological criteria applied for defining lumbar spinal stenosis has been published to date. A structured review focusing on clinical eligibility criteria was recently published showing a high degree of variability in inclusion criteria between studies [8]. The finding of our study, focusing on radiological criteria, strengthens the conclusion from Genevay [8] that there is a need for a consensus on criteria to define and classify lumbar spinal stenosis.

A vague definition of an illness and imprecise criteria to either rule-in or rule-out an illness, as a consequence of that, poses a major problem on performing research in patients with such a disorder. It limits the accurate description of patients enrolled in a study and therefore confines the interpretation and applicability of the obtained results in medical practice. The primary goal of clinical studies is to gain results needed to inform physicians about the intended and adverse effects of different treatment modalities. Our findings indicate shortcomings in defining and classifying lumbar spinal stenosis resulting in imprecise and variable definitions of inclusion criteria in studies evaluating the natural course of the illness or the effect of different treatment modalities. This lack of distinct criteria impairs the interpretation of the study results and the comparability of the findings between different studies.

Two questions are of particular importance concerning diagnosis in patients with clinically suspected lumbar spinal stenosis: First, can a lumbar spinal stenosis be verified by radiological measures? Second, if stenosis is verified by an imaging procedure, are the symptoms and clinical signs caused by the identified somatic anomaly? There is a need to formulate a code of practice, based on shared expert's belief, to set measurement points in the lumbar spine to describe and quantify structural anomalies and in a further step to establish norm- and cut-off values. The aim of further studies might be the development of valid methods to assess the relationship between structural anomalies, symptoms and clinical signs. A difficulty to assess the association of clinical manifestations and anatomical anomalies is the fact that there is no simple reference test for lumbar spinal stenosis. The most obvious reference standard, at least in patients undergoing surgery, could be improvement after surgery. However, surgery at the lumbar spine might have an inadvertent beneficial effect on other mechanical pain generators. Innovative methods have to be developed to overcome these problems.

Although a thorough search in Medline, Embase and the Cochrane Library was performed papers reporting on descriptors of spinal stenosis different from those included in the list we present may have been missed. However, in the primary studies, included in the systematic reviews no additional descriptors not already found in the first search were identified. This indicates that relevant radiologic descriptors of lumbar spinal stenosis have most probably not been missed. A shortcoming of this review is the sole focus on quantitative parameters. Beside them qualitative parameters, e.g., disc protrusion or hypertrophic facet joint degeneration, are used to describe abnormalities in the lumbar spine.

Lumbar spinal stenosis is a common disorder and the most frequent indication for lumbar spine surgery in the elderly. Due to the demographic changes the number of patients with this disorder will increase. There is a need for a consensus among experts on well defined, unambiguous radiological and clinical criteria to define lumbar spinal stenosis. The criteria, reported in this paper, can be used as a source for the development of radiological criteria.

## Conclusions

There is a need for consensus on well-defined, unambiguous radiological criteria to define and characterize lumbar spinal stenosis in order to improve diagnostic accuracy and to formulate reliable inclusion criteria for clinical studies.

## Competing interests

The authors declare that they have no competing interests.

## Authors' contributions

JS has been involved in the conception, acquisition and interpretation of data and was involved in writing the manuscript, SR has been in invovled acquisition and interpretation of data and was involved in writing the manuscript, RG made substantial contributions in the interpretation of data and was involved in writing the manuscript, and JH has been involved in the conception, acquisition and interpretation of data and was involved in writing the manuscript. All authors have given final approval of this version of the manuscript.

## Pre-publication history

The pre-publication history for this paper can be accessed here:

http://www.biomedcentral.com/1471-2474/12/175/prepub

## Supplementary Material

Additional file 1 **Search strategy in Medline**. Search terms, combination of the terms and number of identified publications are reported. Click here for file

Additional file 2 **Radiologic descriptors applied in the original studies**. Radiologic descriptors form the the 20 primary studies comparing various treatment modalities in patients with lumbar spinal stenosis included in four systematic reviews. Click here for file
