# Supplementary material for: Quantitative radiologic criteria for the diagnosis of lumbar spinal stenosis: a systematic literature review
Source: BMC Musculoskelet Disord. 2011 Jul 28;12:175. doi: 10.1186/1471-2474-12-175 (PMC3161920; doi:10.1186/1471-2474-12-175)
Supplement: Additional file 1 — Search strategy in Medline. Search terms, combination of the terms and number of identified publications are reported. [file 1471-2474-12-175-S1.DOC]

|  | search terms | number of publications |
| --- | --- | --- |
| 1 | (spin* adj5 stenos*).mp. [mp=title, original title, abstract, name of substance word, subject heading word, unique identifier] | 4372 |
| 2 | exp Spinal Stenosis/ | 3161 |
| 3 | 1 or 2 | 4372 |
| 4 | (magnetic* adj5 imag*).mp. [mp=title, original title, abstract, name of substance word, subject heading word, unique identifier] | 239715 |
| 5 | exp Magnetic Resonance Imaging/ | 221039 |
| 6 | Tomography Scanners, X-Ray Computed/ | 1338 |
| 7 | tomography.mp. | 339601 |
| 8 | 4 or 5 or 6 or 7 | 521856 |
| 9 | (sensitivity or specificity).mp. [mp=title, original title, abstract, name of substance word, subject heading word, unique identifier] | 992690 |
| 10 | exp "Sensitivity and Specificity"/ | 302698 |
| 11 | ((accurate or specific) adj3 (identification* or finding* or measurement* or testing*)).tw. | 26739 |
| 12 | ((diagnos* or test* or measur*) adj5 accura*).mp. [mp=title, original title, abstract, name of substance word, subject heading word, unique identifier] | 74857 |
| 13 | 9 or 10 or 11 or 12 | 1132863 |
| 14 | 3 and 8 and | 13 146 |
| 15 | limit 14 to (english or german) | 136 |
