# Supplementary material for: Quantitative radiologic criteria for the diagnosis of lumbar spinal stenosis: a systematic literature review
Source: BMC Musculoskelet Disord. 2011 Jul 28;12:175. doi: 10.1186/1471-2474-12-175 (PMC3161920; doi:10.1186/1471-2474-12-175)
Supplement: Additional file 2 — Radiologic descriptors applied in the original studies. Radiologic descriptors form the the 20 primary studies comparing various treatment modalities in patients with lumbar spinal stenosis included in four systematic reviews. [file 1471-2474-12-175-S2.DOC]

| **Systematic review** | **Primary studies** | **Site of stenosis** | **Site of measurement(levels)** | **Definition of stenosis (cut-off values)** | **Imaging procedure** |
| --- | --- | --- | --- | --- | --- |
| **Aalto** |  |  |  |  |  |
|  | Amundsen [2] | Not reported | Not reported | Radiologic signs of compression on the clinically afflicted nerve root(s) The compression should not primarily be caused by a bulging or herniated intervertebral disc, a neoplasm (tumor), or inflammatory process (abscess). | Plain radiography, Myelography, and CT |
|  | Herkowitz [3] | Not reported | Not reported | Constriction of dural sac | Myelogram, myeloCT, MRI |
|  | Iversen [4] | Not reported | Not reported | Compression of cauda equina or nerve root(s) | CT, MRI, Myelography |
|  | Jönsson [5] | Central stenosis | Not reported | Vertebral slipping | conventional x-rays |
|  | Jönsson [6] | Lateral stenosis | Not reported | Medial dislocation of nerve root caused by the facet joint, and a contrast filling defect of the root sleeve distal to the lateral recess  Narrow lateral recess due to congenital abnormality or acquired hypertrophy of the superior articular facet  Reduction of the amount of perineural fat  entrance zone on sagittal images scrutinized for nerve root affliction | Myelography  CT, MRI  MRI |
|  | Jönsson[7] | Central stenosis  Lateral stenosis | Lumbar vertebra 5 | Compression of the dural sac with or without recess-stenosis  Bony compression of single nerve root without reduction of the area of spinal canal | Myelography, CT, MRI |
|  | Katz [8, 9] | Not reported | Not reported | Compression of cauda equina or exiting nerve roots by ligamentum flavum, facet joints, osteophytes or disc material | Myelography, CT, myeloCT, MRI |
|  | Kleeman [10] | Not reported | Not reported | Compressive canal stenosis with or without lateral recess stenosis | MRI, CT, Myelogram |
|  | McGregor [11, 12] | Central or lateral stenosis | Not reported | Nerve root compression as a result of degenerative changes | MRI |
|  | Sato [13] | Not reported | Not reported | five grades of spinal stenosis:  1 Normal  2 Root sleeve deficit  3 Hourglass stenosis  4 Incomplete block  5 Complete block | Myelography |
|  | Yukawa [14] | Central stenosis | cross-sectional area at midpoint of each inter-vertebral level | 70 -100 mm² (moderate)  < 70 mm² (severe) | MRI, myeloCT |
| **Coronado** [**15]** |  |  |  |  |  |
|  |  |  |  |  |  |
|  | Eskola [16] | Central stenosis | Sagittal diameter of spinal canal | < 10 mm | Myelography |
|  | Porter [17] | Not reported | Not reported | Positive radiculogramm showing encroachment of the dural sac  Bony or soft tissue encroachment of the root canal | Myelography  CT |
|  | Tafazal [18] | Central stenosis diameter | Mid-sagittal diameter | ≤ 13 mm | MRI |
|  |  |  |  |  |  |
| **Gibson** [**19]** |  |  |  |  |  |
|  | Amundsen [1] | Not reported | Not reported | Radiologic signs of compression on the clinically afflicted nerve root(s) The compression should not primarily be caused by a bulging or herniated intervertebral disc, a neoplasm (tumor), or inflammatory process (abscess). | Plain radiography, Myelography, and CT |
|  | Grob [20] | Central stenosis | Mid-sagittal diameter of spinal canal | < 11 mm | conventional radiography |
|  | Herkowitz [3] | Not reported | Not reported | Constriction of dural sac | Myelogram, myeloCT, MRI |
| **Genevay** |  |  |  |  |  |
|  |  |  |  |  |  |
|  | Cavusoglu [22] | Not reported | Not reported | Evidence of degenerative lumbar stenosis (neurologic  compression by hypertrophied (infolded) ligamentum flavum,  osteophytic facet joints, and annular bulging) | not reported |
|  | Hallett [23] | Foraminal stenosis | Not reported | Intraforaminal or extraforaminal nerve root compromising,  in association with single-level degenerative disc  disease | MRI |
|  | Tafazal [18] | Central stenosis | Mid-sagittal diameter | ≤ 13 mm | MRI |
|  | Whitman [24] | central or foraminal stenosis | Not reported | Findings consistent with LSS (evidence of compression of lumbar spinal nerve root(s) by degenerative lesions of the facet joint, disc, and/or ligamentum flavum) | MRI |

References:

1. Aalto T, Malmivaara A, Kovacs F, Herno A, Alen M, Salmi L, Kröger H, Andrade J, Jiménez R, Tapaninaho A, Turunen V, Savolainen S, Airaksinen O: **Preoperative predictors for postoperative clinical outcome in lumbar spinal stenosis: systematic review**. *Spine (Phila Pa 1976)* 2006, **31**(18):E648-663.

2. Amundsen T, Weber H, Nordal H, Magnaes B, Abdelnoor M, Lilleâs F: **Lumbar spinal stenosis: conservative or surgical management?: A prospective 10-year study**. *Spine (Phila Pa 1976)* 2000, **25**(11):1424-1435.

3. Herkowitz H, Kurz L: **Degenerative lumbar spondylolisthesis with spinal stenosis. A prospective study comparing decompression with decompression and intertransverse process arthrodesis**. *J Bone Joint Surg Am* 1991, **73**(6):802-808.

4. Iversen M, Daltroy L, Fossel A, Katz J: **The prognostic importance of patient pre-operative expectations of surgery for lumbar spinal stenosis**. *Patient Educ Couns* 1998, **34**(2):169-178.

5. Jönsson B: **Vertebral slipping after decompression for spinal stenosis**. *Acta Orthop Scand Suppl* 1993, **251**:76-77.

6. Jönsson B, Strömqvist B: **Decompression for lateral lumbar spinal stenosis. Results and impact on sick leave and working conditions**. *Spine (Phila Pa 1976)* 1994, **19**(21):2381-2386.

7. Jönsson B, Strömqvist B: **Motor affliction of the L5 nerve root in lumbar nerve root compression syndromes**. *Spine (Phila Pa 1976)* 1995, **20**(18):2012-2015.

8. Katz J, Lipson S, Brick G, Grobler L, Weinstein J, Fossel A, Lew R, Liang M: **Clinical correlates of patient satisfaction after laminectomy for degenerative lumbar spinal stenosis**. *Spine (Phila Pa 1976)* 1995, **20**(10):1155-1160.

9. Katz J, Stucki G, Lipson S, Fossel A, Grobler L, Weinstein J: **Predictors of surgical outcome in degenerative lumbar spinal stenosis**. *Spine (Phila Pa 1976)* 1999, **24**(21):2229-2233.

10. Kleeman T, Hiscoe A, Berg E: **Patient outcomes after minimally destabilizing lumbar stenosis decompression: the "Port-Hole" technique**. *Spine (Phila Pa 1976)* 2000, **25**(7):865-870.

11. McGregor A, Hughes S: **The evaluation of the surgical management of nerve root compression in patients with low back pain: Part 1: the assessment of outcome**. *Spine (Phila Pa 1976)* 2002, **27**(13):1465-1470.

12. McGregor A, Hughes S: **The evaluation of the surgical management of nerve root compression in patients with low back pain: Part 2: patient expectations and satisfaction**. *Spine (Phila Pa 1976)* 2002, **27**(13):1471-1476.

13. Sato K, Kikuchi S: **Clinical analysis of two-level compression of the cauda equina and the nerve roots in lumbar spinal canal stenosis**. *Spine (Phila Pa 1976)* 1997, **22**(16):1898-1903.

14. Yukawa Y, Lenke L, Tenhula J, Bridwell K, Riew K, Blanke K: **A comprehensive study of patients with surgically treated lumbar spinal stenosis with neurogenic claudication**. *J Bone Joint Surg Am* 2002, **84-A**(11):1954-1959.

15. Coronado-Zarco R, Cruz-Medina E, Arellano-Hernandez A, Chavez-Arias D, Leon-Hernandez SR: **Effectiveness of calcitonin in intermittent claudication treatment of patients with lumbar spinal stenosis: a systematic review**. *Spine (Phila Pa 1976)* 2009, **34**(22):E818-822.

16. Eskola A, Pohjolainen T, Alaranta H, Soini J, Tallroth K, Slätis P: **Calcitonin treatment in lumbar spinal stenosis: a randomized, placebo-controlled, double-blind, cross-over study with one-year follow-up**. *Calcif Tissue Int* 1992, **50**(5):400-403.

17. Porter R, Miller C: **Neurogenic claudication and root claudication treated with calcitonin. A double-blind trial**. *Spine (Phila Pa 1976)* 1988, **13**(9):1061-1064.

18. Tafazal S, Ng L, Sell P: **Randomised placebo-controlled trial on the effectiveness of nasal salmon calcitonin in the treatment of lumbar spinal stenosis**. *Eur Spine J* 2007, **16**(2):207-212.

19. Gibson JN, Waddell G: **Surgery for degenerative lumbar spondylosis: updated Cochrane Review**. *Spine (Phila Pa 1976)* 2005, **30**(20):2312-2320.

20. Grob D, Humke T, Dvorak J: **Degenerative lumbar spinal stenosis. Decompression with and without arthrodesis**. *J Bone Joint Surg Am* 1995, **77**(7):1036-1041.

21. Genevay S, Atlas SJ, Katz JN: **Variation in eligibility criteria from studies of radiculopathy due to a herniated disc and of neurogenic claudication due to lumbar spinal stenosis: a structured literature review**. *Spine (Phila Pa 1976)* 2010, **35**(7):803-811.

22. Cavuşoğlu H, Kaya R, Türkmenoglu O, Tuncer C, Colak I, Aydin Y: **Midterm outcome after unilateral approach for bilateral decompression of lumbar spinal stenosis: 5-year prospective study**. *Eur Spine J* 2007, **16**(12):2133-2142.

23. Hallett A, Huntley J, Gibson J: **Foraminal stenosis and single-level degenerative disc disease: a randomized controlled trial comparing decompression with decompression and instrumented fusion**. *Spine (Phila Pa 1976)* 2007, **32**(13):1375-1380.

24. Whitman J, Flynn T, Childs J, Wainner R, Gill H, Ryder M, Garber M, Bennett A, Fritz J: **A comparison between two physical therapy treatment programs for patients with lumbar spinal stenosis: a randomized clinical trial**. *Spine (Phila Pa 1976)* 2006, **31**(22):2541-2549.
